# Apoptotic markers in protozoan parasites

**DOI:** 10.1186/1756-3305-3-104

**Published:** 2010-11-09

**Authors:** Antonio Jiménez-Ruiz, Juan Fernando Alzate, Ewan Thomas MacLeod, Carsten Günter Kurt Lüder, Nicolas Fasel, Hilary Hurd

**Affiliations:** 1 Departamento de Bioquímica y Biología Molecular, Universidad de Alcalá, 28871 Alcalá de Henares, Madrid, Spain; 2 Grupo de Parasitología, Departamento de Microbiología y Parasitología, Facultad de Medicina, Universidad de Antioquia. Carrera 51D # 62-29, Medellín, Colombia; 3 Division of Pathway Medicine, School of Biomedical Sciences, College of Medicine and Veterinary Medicine, University of Edinburgh, 1Summerhall Square, Edinburgh EH9 1QH, UK; 4 Institute for Medical Microbiology, Georg-August-University, Kreuzbergring 57, 37075 Göttingen, Germany; 5 Department of Biochemistry, Univerisity of Lausanne, 1066 Epalinges, Switzerland; 6 Institute for Science and Technology in Medicine, Centre for Applied Entomology and Parasitology, School of Life Sciences, Keele University, Staffordshire, ST5 5BG, UK

## Abstract

The execution of the apoptotic death program in metazoans is characterized by a sequence of morphological and biochemical changes that include cell shrinkage, presentation of phosphatidylserine at the cell surface, mitochondrial alterations, chromatin condensation, nuclear fragmentation, membrane blebbing and the formation of apoptotic bodies. Methodologies for measuring apoptosis are based on these markers. Except for membrane blebbing and formation of apoptotic bodies, all other events have been observed in most protozoan parasites undergoing cell death. However, while techniques exist to detect these markers, they are often optimised for metazoan cells and therefore may not pick up subtle differences between the events occurring in unicellular organisms and multi-cellular organisms.

In this review we discuss the markers most frequently used to analyze cell death in protozoan parasites, paying special attention to changes in cell morphology, mitochondrial activity, chromatin structure and plasma membrane structure/permeability. Regarding classical regulators/executors of apoptosis, we have reviewed the present knowledge of caspase-like and nuclease activities.

## Review

Recently there has been a move to clarify the classification of cell death [1] and to set up guidelines for cell death assays in eukaryotes [2]. While these events have been studied in metazoans for more than two decades, the presence of apoptosis-like processes in protozoans was not widely accepted when the first papers on events similar to metazoan apoptosis were published 15 years ago [3,4]. Therefore, considering the diversity of parasites and conditions for their study, there is an urgent need to define the markers, both cellular and molecular, which are the most accurate to assign as apoptosis-like events that occur during the death process of protozoan parasites. Defining the events that occur during death are of paramount importance to this field of study, since so far there is no definitive guide as to what happens in each parasitic protozoan when it undergoes this process, nor is it clear whether similar cell death phenotypes are exhibited by all taxa of unicellular organisms. Moreover, some of these assays are likely to be positive in cells undergoing different types of cell death. This fact stresses the relevance of using several markers simultaneously to confirm an apoptotic phenotype.

According to the Nomenclature Committee on Cell Death (NCCD) [1], the term apoptosis describes a specific morphological aspect of cell death. Cellular alterations during this process include rounding-up of the cell, reduction of cellular volume (pyknosis), chromatin condensation, nuclear fragmentation, plasma membrane blebbing and, if in the right context, engulfment by resident phagocytes. Little or no ultrastructural modifications of cytoplasmic organelles are observed during apoptosis. On the contrary, necrotic cell death is morphologically characterized by a gain in cell volume (oncosis), swelling of organelles, plasma membrane rupture and subsequent loss of intracellular contents. In the absence of common biochemical markers, early plasma membrane permeabilization is considered the main hallmark of necrosis. Otherwise, necrotic cell death is still largely identified in negative terms by the absence of apoptotic or autophagic markers.

Following these guidelines, several changes seem to be useful to define whether cells die following an apoptotic or a necrotic death. From a morphological point of view, a pyknotic cell should be considered to be in the process of apoptotic cell death. Regarding biochemical markers, detection of DNA fragmentation or caspase activation while maintaining plasma membrane integrity clearly identify apoptotic cells. Mitochondrial membrane potential (ΔΨ_m_) dissipation in the context of a non-ruptured plasma membrane and opening of the mitochondrial membrane pore are also biochemical features of apoptotic cells that are not present in necrotic cells [1]. The use of ΔΨ_m_-sensitive probes or the identification of the subcellular localization of mitochondrial markers such as cytochrome c or endonuclease G are also very useful tools to identify an apoptotic phenotype. Based on this consensus, the purpose of this review is to critically analyze the use of these markers in parasitic protozoa and give some recommendations about how they should be used. Throughout the text we will use the term "apoptotic phenotype" to define what is observed using the markers under discussion, although this term may not be universally accepted as a descriptor for cell death in protists. While markers and/or molecular correlates of apoptosis have indeed been observed in the protozoan parasites analyzed so far [5], the biochemical pathways that precede their appearance have not yet been clarified.

Although the first descriptions of apoptotic phenotypes in protozoa were made in *Trypanosoma cruzi *[3] and *Trypanosoma brucei *[4], *Leishmania *has been used as the main model organism to study them and define possible cell death markers. Studies have been made of the different life stages of the parasites and a wide arsenal of drugs or stress conditions have been used to induce death. Lectins such as ConA [4,6] were amongst the first compounds shown to induce the expression of apoptotic markers in *T. brucei*. Since the turn of the century several other compounds and conditions (reviewed in Duszenko et al., [7]) including prostaglandins and high density culture have also been reported to stimulate the appearance of apoptotic phenotypes. Cell death can also be induced by RNA interference of essential genes [8]. This tool, not possible in *Leishmania *[9], could be quite instrumental to induce or block cell death in defined conditions. Of the few studies of the manifestation of apoptotic markers in the malaria parasites, the majority have been made with the intra-erythrocytic asexual stages of *Plasmodium falciparum *with observations made *in vitro*. Observations of drug-induced cell death have not been consistent from parasite strain to strain and conclusions concerning definitions of cell death type are controversial. Markers of apoptosis-like cell death have also been observed in the motile zygote, the ookinete, which develops in the midgut lumen of the mosquito both *in vivo *and *in vitro*. Several cellular processes that resemble apoptosis in metazoans have also been described in tachyzoites of the apicomplexan parasite *T. gondii *[10].

Therefore, because of the extensive work of many groups on *Leishmania*, the most relevant data concerning this parasite will be presented for each category of marker and, where possible, data from *Trypanosoma, Plasmodium *and *Toxoplasma *will also be reviewed. Table 1 summarizes the most relevant assays developed in these species. However, we should keep in mind that, in the reported studies, some parameters could be different: density of the parasite culture, life cycle stage or simply possible difference between species or strains.

**Table 1:** Summary of the markers used to analyze apoptotic phenotypes in protozoan parasites upon different death stimuli. *Plasmodium falciparum *data refer to erythrocyte stages in culture.

*Parasite*	marker	Death stimulus
***Leishmania donovani***	PS exposure	novobiocin [22]; miltefosine [23]; respiratory chain inhibitors [32]

	ΔΨm changes	stationary phase [31]; edelfosine [30]

	Cytochrome C release	novobiocin [22]; miltefosine [43]; withaferin A [33]

	Caspase-like activity	stationary phase [31]; amphotericin b [31]; novobiocin [22]; miltefosine [23]; camptothecin [65]; flavonoids [24]

	DNA degradation	stationary phase [31]; novobiocin [22]; miltefosine [23]; respiratory chain inhibitors [32]; H_2_O_2_; [62]

		

***Leishmania infantum***	PS exposure	heat shock [12]

	ΔΨm changes	edelfosine [30]; heat shock [12]

	Caspase-like activity	edelfosine [30]; heat shock [12]

	DNA degradation	edelfosine [30]; heat shock [12]

		

***Leishmania major***	Caspase-like activity	serum deprivation/stationary phase [34]

	DNA degradation	serum deprivation/stationary phase [34]

		

***Leishmania amazonensis***	PS exposure	metacyclogenesis [66]

	DNA degradation	nitric oxide (NO) [61]

		

***Leishmania mexicana***	Caspase-like activity	serum deprivation/stationary phase [34]

	DNA degradation	serum deprivation/stationary phase [34]

		

***Toxoplasma gondii***	Chromatin condensation	sodium nitroprusside (NO) [10]

	DNA degradation	sodium nitroprusside (NO) [10]

	Hypoploid nuclei	sodium nitroprusside (NO) [10]

	PS exposure	intraperitoneal death in vivo in mice [28]

		

***Trypanosoma brucei (s.l.)***	PS exposure	high density culture [25]; prostaglandins [26,41]; persistant ER stres [8] quercetin [73]

	ΔΨm changes	prostaglandins [26,41]; modified bovine host defense peptide [40]; persistant ER stres [8]

	Cytochrome C release	expression of proapoptotic Bax protein [44]

	DNA degradation	high density culture [25]; prostaglandins [26,41]; persistant ER stres [8]; lectin ConA [4]; H_2_O_2 _[67]

		

***Plasmodium berghei***	PS exposure	ookinete stage in vitro [17,29]; nitric oxide in vitro [36]

	ΔΨm changes	ookinete stage in vitro [29]; nitric oxide in vitro [36]

	Caspase-like activity	ookinte stage in vitro and in vivo [17]; ookinte stage in vivo [29]; nitric oxide in vitro [36]

	DNA degradation	ookinete stage in vitro [17,29]

	chromatin condensation	ookinte stage in vitro and in vivo [17]; ookinte stage in vivo [29]; nitric oxide in vitro [36]; L-dopa in vitro [36]

		

***Plasmodium falciparum***	ΔΨm changes	chloroquine [14,37]; staurosporine [37]; bilirubin [38];, atovaquone [39]; heat shock [15]

	Caspase-like activity	chloroquine [14,37]; staurosporine [37]; 4-hydroxytamoxifen [37]; bilirubin [38]

	DNA degradation	cholorquine [13,14,37]; etoposide [14]; staurosporine [37]; 4-hydroxytamoxifen [37]

		

***Trichomonas vaginalis***	PS exposure	etoposide, doxorubicin [74]

	ΔΨm changes	etoposide, doxorubicin [74]

	DNA degradation	etoposide, doxorubicin [74]

		

***Blastocystis hominis***	PS exposure	cytotoxic monoclonal antibody [75]; metronidazole [76]; staurosporine [77]

	ΔΨm changes	cytotoxic monoclonal antibody [78]

	Caspase-like activity	cytotoxic monoclonal antibody [78]

	DNA degradation	cytotoxic monoclonal antibody [79]; metronidazole [76]; staurosporine [77]

	chromatin condensation	staurosporine [77]

		

***Entamoeba histolytica***	Morphological changes	G418 [80]

	chromatin condensation	G418 [80]

	DNA degradation	G418 [80]

		

***Giardia lamblia***	PS exposure	metronidazole, H_2_O_2_; [81]

	DNA degradation	metronidazole, H_2_O_2_; [81]

### Morphological changes

**Figure 1:**
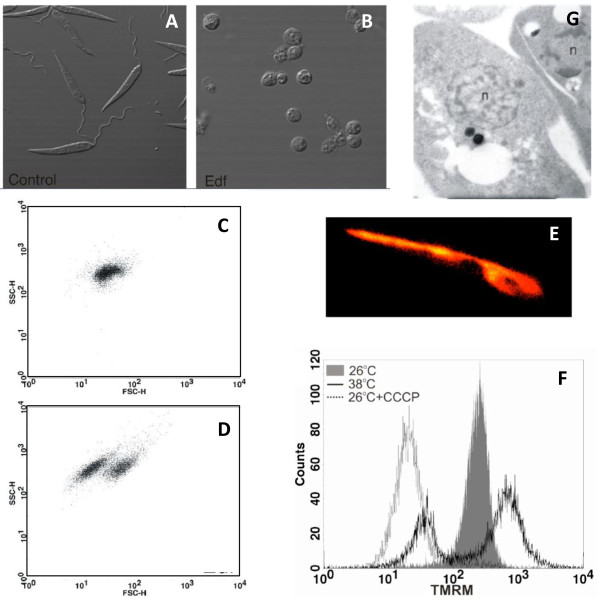
**Markers of apoptosis in *Leishmania infantum *promastigotes grown in vitro**. **A**. Elongated *L. infantum *promastigotes grown *in vitro *under control conditions. **B**. Rounded *L. infantum *promastigotes grown *in vitro *in the presence of edelfosine. **C**. Flow cytometric analysis showing a bi-parametric plot of the forward and side scatter properties of *L. infantum *promastigotes grown in vitro under control conditions. **D**. Flow cytometric analysis showing a bi-parametric plot of the forward and side scatter properties of *L. infantum *promastigotes grown *in vitro *in the presence of edelfosine. **E**. *L. infantum *promastigotes labelled with the potentiometric probe TMRM showing specific staining of the mitochondrion. **F**. Flow cytometric analysis showing a mono-parametric plot of the TMRM-derived fluorescence emitted by *L. infantum *promastigotes grown at 26°C, 38°C and in the presence of the mitochondrial uncoupler CCCP. **G**. Electron microscopic image of two *L. infantum *promastigotes grown at 38°C. n = nuclei.

Apoptosis in higher eukaryotes involves a series of biochemical events leading to characteristic changes in cell morphology and subsequent death. During the execution phase of apoptosis, the proteolytic activity of caspases disrupts the cytoskeleton, which is no longer able to maintain cell shape and, as a consequence of the homogeneous osmotic pressure, all cells become rounded. Many protozoan parasites are clearly identified by their characteristic shapes that, similarly to metazoans, are lost during cell death. Changes in cell shape can be clearly observed under the microscope (compare the elongated shape of untreated cultured *Leishmania *promastigotes with that of edelfosine-treated ones, Figure 1A and 1B).

Apoptotic death in metazoans is also characterized by cell shrinkage [11], which can be analysed either by microscopic observation or by flow cytometry based on the changes in the scattering properties of the cells when the laser beam is directed to them. Forward scatter values (FSC) correlate with cell volume and can be used to determine changes in it. Bi-parametric plots showing the forward (FSC) and side scatter (SSC) properties of the cell population are very useful to detect changes in cell volume and have been successfully employed to detect cell shrinkage associated with death in protozoan parasites such as *Leishmania *(Figure 1C and 1D) [12]. Some of the earliest reports of apoptotic phenotypes in *Trypanosoma *concentrated on morphological changes. For instance Ameisen et al. [3] and Welburn et al. [4] described cytoplasmic vacuolisation and margination, extensive membrane blebbling and condensation of nuclear chromatin in *T. cruzi *and *T. brucei *respectively.

In unfavourable culture conditions or following drug treatment, condensed, so called 'crisis forms', of the intra-erythrocytic stage of *P. falciparum *(3D7 strain) appear. Although these forms have no precise definition they have been hypothesised to be undergoing apoptosis [13] and their formation can be blocked by the caspase-inhibitor Z-VAD-FMK [14]. In contrast, no drug-induced cell shrinkage was reported in the CSC-1 strain, though ultrastructural analysis additionally revealed food vacuole swelling and lysis [15]. Even though shrinkage in cells with non-compromised plasma membranes is one of the main morphological markers of apoptosis [1], necrotic cells also diminish their volume once the plasma membrane breaks, so these morphological changes could also be indicators of necrosis and more efforts need to be made to distinguish between morphological changes between different forms of cell death in *Plasmodium*. Vacuole formation in the chloroquine-resistant Brazilian PSS1 strain was regarded as evidence of autophagy, although no other autophagic markers were detected [16]. No obvious cell shrinkage or other change in cell shape has been detected in dying ookinetes of the rodent malaria *Plasmodium berghei*, suggesting no major disruption of the cytoskeleton occurs when other markers of apoptosis are exhibited [17]. Distinct morphological changes that resemble apoptosis in metazoans have been also described in tachyzoites of the apicomplexan parasite *T. gondii *following treatment with the nitric oxide (NO) donor sodium nitroprusside (SNP) [10]. Rounding-up of the normally elongated tachyzoites and cell shrinkage were most prominent as determined by transmission electron microscopy [10]. Since it coincided with the occurrence of chromatin condensation (see also below) these alterations resembled apoptotic cell death in metazoans. Unfortunately, cell sizes of *T. gondii *treated or not with SNP have not been analysed by flow cytometry as described above. It thus remains unclear to what extent the parasites shrink following exposure to NO.

### Plasma membrane alterations

Two main alterations in the plasma membrane have been described during cell death processes: phosphatidyl serine (PS) externalization and permeabilization to propidium iodide (PI). Under normal physiologic conditions, PS is predominantly located in the inner leaflet or cytosol-facing part of the plasma membrane. Upon initiation of apoptosis, PS loses its asymmetric distribution in the phospholipid bilayer and is translocated to the extracellular membrane leaflet where it identifies cells as targets for phagocytosis. PS presentation in the outer membrane face is easily analyzed using labelled Annexin V. This protein binds to PS as part of its biological activity [18]. Accordingly, non-permeabilized cells are incubated with labelled (often FITC-labelled) Annexin V, which only stains the cells when PS has already been translocated to the outer leaflet.

An early event of apoptosis in metazoans is PS presentation in the outer face of the plasma membrane [19]. At this stage, the plasma membrane is still able to exclude viability dyes such as PI, so single staining with Annexin V serves as an early marker of apoptosis. In late stage apoptosis the membrane may lose its integrity allowing Annexin V to access the interior of the cell and stain PS still located in the inner membrane leaflet. The absence of PI staining signals membrane integrity and ensures that Annexin V is only binding the cells through the PS located in the external membrane surface [20]. However, it must be pointed out that Annexin V can also bind anionic phospholipids other than PS [21]. Additional markers described to bind PS such as protein S and PS-specific monoclonal antibodies may be used to ensure the presence of PS in the outer membrane face [21].

An important aspect to be considered when analyzing PS/PI staining of parasites is DNA degradation. For instance, most of the classical laboratory strains of *Leishmania *show very rapid degradation of their DNA under almost any death stimuli. Accordingly, the fast PI permeabilization usually observed in these parasites can be underscored as a consequence of DNA degradation: cells may become PI negative shortly after cell death as a consequence of a reduction in the amount of DNA inside the cells (Jiménez-Ruiz; unpublished results). For this reason, PS externalization analyses in parasites should be designed to include the observation of samples at different times after death induction in order to follow the movement of the population from PS negative/PI negative (living cells) to PS positive/PI negative (canonical apoptotic marker) and lastly to PS positive/PI positive (late apoptotic marker). Further incubation will render most of the population PS positive/PI negative again as a consequence of DNA degradation which can easily mislead observers to consider that these cells express a canonical apoptotic phenotype. Probably because of the difficulty to obtain canonical PS positive/PI negative populations experienced with several parasite species, in many published manuscripts the authors only present the results obtained after Annexin V labelling either by fluorescent microscopy or by flow cytometry.

PS presentation has been reported in *Leishmania *under diverse cell death inducers such as treatment with novobiocin [22], miltefosine [23], luteolin [24] or heat shock [12]. PS exposure has also been observed in *T. brucei *following exposure to a variety of stimuli including high density culture [25], prostaglandins of the J series [26] and persistent endoplasmic reticulum (ER) stress [8]. At high density culture, Tsuda et al. showed that after 24 hours the majority of cells were Annexin V positive and PI negative [25]. Incubation of bloodstream forms of *T. brucei *with prostaglandins from the J series for between two and six hours caused the appearance of PS on the outer membrane; the process was inhibited by cyclohexmide suggesting that active protein synthesis is required [26]. Goldschmidt et al. [8] induced PS translocation to the outer membrane of procyclic forms by persistent ER stress with dithiothreitol. This was achieved two days after the silencing of ER translocation machinery by RNAi, followed by 12 hours exposure to dithiothreitol. In each case trypanosomes became permeable to PI after a further 12 hours. As discussed above in relation to the timeframe for PS flipping and DNA degradation, the exposure of trypanosomes to dithiothreitol over time showed that they firstly expressed PS on their surface before becoming permeable to PI. It is interesting to highlight that the presence of PS-positive *Leishmania *promastigotes has been shown to inhibit the host macrophage inflammatory response, which allows efficient *in vitro *and *in vivo *infections by the PS-negative parasites [27].

Externalization of PS to the outer leaflet of the cell membrane appears to also occur in *Toxoplasma *[28]. Importantly, a significant proportion of PS-positive cells were detected among parasites that had been freshly isolated from the peritoneal cavity of infected mice suggesting that it may occur during infection in vivo [28]. Since the PS-positive parasites from the peritoneum of mice were predominantly PI-negative they clearly met an important characteristic of the apoptotic phenotype. However, it has to be mentioned that the transition from Annexin V-positive/PI-negative to Annexin V-positive/PI-positive after isolation from infected mice was not further analysed. In addition, the occurrence of other apoptotic markers in *Toxoplasma *parasites displaying PS on their surface also awaits clarification.

**Figure 2:**
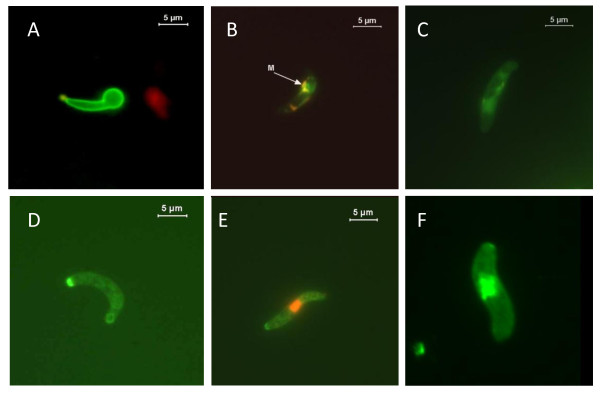
**Markers of apoptosis detected in *Plasmodium berghei *ookinetes grown *in vitro***. **A**. A developing ookinete (retort) exhibiting phosphatidylserine translocation to the outer membrane surface, stained with Annexin V (taken from Arambage et al. [29]). **B **and **C**. Ookinetes following a JC-1 assay: the mitochondrial membrane potential is intact in B (orange coloured aggregates (m)) and has been lost in C. **D **and **E**. Ookinetes following incubation with the caspase substrate fam-VAD-FMK (CaspaTag), D = caspase +ve/PI -ve, E = caspase +ve/PI +ve. **F. **An ookinete stained positive for DNA fragmentation using the TUNEL assay (provided by L. Politt).

The various membranes surrounding the intra-erythrocyte stage of *Plasmodium *complicate the identification of any PS translocation detection with Annexin V as isolation of the parasites from these surrounding membranes may not be entirely successful; producing results that are not trustworthy. However, ookinetes are not intracellular stages and PS positive/PI negative parasites have been observed *in vitro *(Figure 2A) [29].

### Mitochondrial alterations

Mitochondria are key players in cell death. Trypanosomatids have a single large mitochondrion and alterations in the mitochondrial function have been studied as one of the markers of cell death in protozoan parasites [12,23,30-34]. Tetramethylrhodamine methyl ester (TMRM) has been successfully used, both in metazoans and in protozoans, to detect changes in mitochondrial transmembrane potential [12,35]. Two important pre-requisites should be taken into consideration before deducing conclusions from the use of this dye: i) specific mitochondrial labelling should be confirmed by fluorescence microscopy and ii) mitochondrial uncouplers such as CCCP (carbonyl cyanide m-chlorophenylhydrazone) should be assayed to ensure that probes are able to detect changes in mitochondrial potential (Figure 1E and 1F) [12].

In many cases mitochondrial depolarization is preceded by a transient hyper-polarization that has often been considered as the last attempt by the cells to avoid death. This effect can be clearly observed in the majority of the population of heat-shocked *Leishmania *promastigotes in Figure 1F.

JC-1 (5,5',6,6'-tetrachloro-1,1',3,3'-tetraethylbenzimidazolylcarbocyanine iodide) is another probe frequently used to detect changes in mitochondrial transmembrane potential. At low concentrations or low membrane potential it exists mainly as a monomer that emits green fluorescence, but at higher concentrations (aqueous solutions above 0.1 μM) or higher potentials JC-1 forms red-fluorescent aggregates that exhibit an emission maximum at 590 nM. The ratio of red to green fluorescence is then an indirect measure of the mitochondrial transmembrane potential that is independent of other factors that may influence single-component fluorescence signals, such as mitochondrial size, shape and density. Despite the value of analyzing this red to green ratio, most of the groups studying parasites just show an increase in the green fluorescence as an indication of mitochondrial depolarization. JC-1 has been used as a probe to detect loss of mitochondrial membrane potential (ΔΨ_m_) in *P. falciparum *and *P. berghei; *in both cases using fluorescence microscopy rather than analyzing red to green ratios (Figure 2B and 2C) [14,29,36]. Incubation of *P. falciparum *erythrocyte stages with both chloroquine and atovaquone increased JC-1 monomers (green staining) in a time and concentration dependent manner but this was significantly lower in a drug resistant form [14]. Using red to green ratios, Ch'ng and colleagues [37] recently confirmed this loss of ΔΨ_m _when *P. falciparum *are exposed to chloroquine in a dose and exposure time dependent manner (Table 2).

**Table 2:** Comparison of assays for the effect of chloroquine (CQ) on ΔΨ_m _in *Plasmodium falciparum*.

*Plasmodium falciparum *strain	Chloroquine concentration	Chloroquine incubation time	Probe	Affect on ΔΨ_m_	Reference
F32 (CQ sensitive)	20 nM	20 min	DiOC_6_(3)	No effect	Nyakeriga et al. [39]

3D7 (CQ sensitive) 7G8 (CQ resistant)	55 nM 1400 nM	18 h 18 h	JC-1 JC-1	Sig. loss Not sig.	Meslin et al. [14]

3D7 (CQ sensitive) 7G8 (CQ resistant) K1 (CQ resistant)	>3 μM >3 μM >12 μM	4 h >8 h >8 h	JC-1 JC-1 JC-1	Sig. loss Sig. loss Sig. loss	Ch'ng et al. [37]

All assays were performed in red blood cell stages. Higher doses and longer incubation times altered the effect of the drug on loss of mitochondrial transmembrane potential (ΔΨ_m_) and different strains of parasite reacted differently.

Permanent loss of ΔΨ_m _was also detected using the cationic probe DiOC_6 _when *P. falciparum *CSC-1 strain was drug treated or subjected to heat shock at 40°C [15]. Bilirubin and actinomycin D also induced a decrease in ΔΨ_m_, in strain NF-54, as detected both by spectrofluorometric analysis (as a ratio of 590 nm/530 nm absorbance) and fluorescence microscopy, the former action being attributed to increased production of ·OH following inhibition of haemozoin formation [38]. In contrast, using the cationic probe DiOC_6_, Nyakeriga et al. [39] were unable to detect a change in ΔΨ_m _when *P. falciparum *strain F 32 (claimed to be chloroquine sensitive) was incubated with either chloroquine or atovaquone and Totino and colleagues used rhodamine staining to detect loss of ΔΨ_m _in a chloroquine-resistant *P. falciparum *PSS1 strain, but regarded this as a general marker for cell death not specific to apoptosis [16]. These conflicting observations may be the result of the use of different experimental protocols. In particular, time of exposure and concentration of drug appears to affect ΔΨ_m _(Table 2).

Recently Haines et al. [40] showed that a modified bovine host defence peptide (BMAP-18) could induce an apoptotic phenotype in *T. brucei*. Immunofluorescence staining with rhodamine 123 showed disruptions to mitochondrial membrane potential without any damage to the plasma membrane of the trypanosome (shown by fluorescein diacetate retention). Loss of rhodamine 123 was followed over a 30-minute timeframe by flow cytometry, with the results clearly showing a decreasing fluorescence over time. When trypanosomes were incubated with higher doses of BMAP-18 the plasma membrane became compromised and death occurred via necrosis. Disruptions to the mitochondrial potential were also observed when trypanosomes were incubated with prostaglandins of the J- [26] and D-series [41] as determined by TMRM staining.

It must be pointed out that the use of DIOC_6 _and rhodamine as fluorescent probes to asses ΔΨ_m _changes in cells is questioned: plasma membrane depolarization in the U937 human cell line causes a change in the intensity of DIOC_6 _staining and addition of the mitochondrial uncoupler FCCP (carbonyl cyanide p-trifluoromethoxy-phenylhydrazone) did not result in changes in the fluorescence emission of rhodamine 123 [42].

One of the hallmarks of apoptosis in mammalian cells is cytochrome c release from the mitochondria to the cytosol, where it binds to the adaptor molecule, apoptotic protease activating factor (Apaf-1), which subsequently activates procaspase-9. Even though protozoan parasites and mammalian cells have diverged hugely during evolution, cytochrome c seems to be a highly conserved protein that allows the antibodies included in several commercial kits designed for use with metazoans to detect it, for example, in *Leishmania*. The results presented by several groups indicate a clear release of this molecule from the mitochondrion to the cytosol after several death-inducing treatments such as novobiocin [22], miltefosine [43] or the protein kinase inhibitor withaferin A [33]. Similarly, cytochrome c was shown to be released from the mitochondrion when the proapoptotic Bax protein was expressed in *T. brucei*. In this case, the antibodies used were generated against peptides from *T. brucei *cytochrome c [44]. No reports of cytochrome c release from mitochondria have been described in *Plasmodium *or in *Toxoplasma*.

Fission of mitochondria has only been followed in a few cases [44]. In *Trypanosoma*, this event occurs after cytochrome c release from the intermembrane space and after mitochondrial membrane depolarization, and can be visualized by confocal microscopy after Mitotracker staining.

Such events affecting the mitochondria should also deplete the cells of ATP, which can be measured by fluorescence using commercial kits [24,45]. However, only a few studies have used ATP levels as a marker of cell death and therefore, ATP depletion measurement as a marker of cell death should be taken with some caution.

### Caspase-like activity

Caspases and the members of the Bcl-2 family are the most relevant regulators of the apoptotic process in metazoans. There is very little information about the possible existence of homologs of the Bcl-2 proteins in protozoan parasites, even though some indirect evidence indicates that they may exist [12,46]. On the other hand, extensive evidence of the existence of caspase-like activities and nuclease activities associated with parasite death processes has been published.

Many groups have reported the activation of proteases able to degrade classical substrates of caspases during cell death in parasites [12,22,23,30,31,34]. Those experiments usually analyze the fluorescence obtained after the proteolytic cleavage of a substrate that liberates a fluorescent group, the peptide DEVD associated with different fluorophores being the substrate most used. Emitted fluorescence is usually analyzed by flow cytometry or fluorometry. Other caspase detection systems utilize fluorescently labeled inhibitors of caspase activity to irreversibly bind to active caspases. However, this limits the sensitivity of the assay since products are not amplified enzymatically. It must be pointed out that even though protease activities have been repeatedly reported during cell death in parasites, they do not seem to be due to real caspases, as no authentic caspase has been identified in protozoans. In *Leishmania*, this activity can be inhibited by E-64, suggesting that these proteases could be similar to cathepsin [34]. Several substrate analogues (DEVD-FMK) are currently used as inhibitors and their ability to decrease the fluorescent signal is considered by some groups as strong evidence of the presence of caspase-like molecules in some parasites. Those results should be analyzed with caution. They do not allow us to conclude that caspase-like activity is present in protozoa until the enzyme has been molecularly characterized. However, it must be pointed out that several groups have been able to prevent the appearance of classical apoptotic markers such as reduction in the transmembrane mitochondrial potential, DNA degradation or PARP cleavage by incubating the cells with these inhibitors [47]. Thus far, we can only conclude that a protease recognizing DEVD or inhibited by DEVD-FMK may be involved in cell death. It has been reported that a number of commonly used small peptide caspase inhibitors efficiently inhibit other cysteine proteases than caspases [48]. The pan-caspase inhibitor VAD-FMK, has been shown to bind and inhibit cathepsin B [48,49]. This inhibition is able to interfere with cell death pathways in mammalian cells [50], which suggests that data based on the use of these inhibitors should be taken with caution because other proteases different to caspases may be participating in these processes. Recent results demonstrate the relevance of cathepsin activity in *Leishmania *cell death [51]. Moreover, these data put some emphasis on a lysosomal cell death pathway based on lysosomal membrane permeabilisation and release of lysosomal enzymes.

Several metacaspases have been described in *Trypanosoma and Leishmania *but their substrate specificity differs to caspases as it seems to be directed to substrates with an arginine or lysine residue in P1 position [52-54]. Metacaspase genes have also been found in the genome of *Plasmodium *[55]. Even though some authors consider that plant and fungal metacaspases lack caspase activity and that they are not responsible for the caspase-like activities detected during their cell death [56], there is evidence that indicates that metacaspases are involved in regulating some death processes in *Leishmania *[53,57]. The use of fluorogenic substrates to detect metacaspase activity associated to cell death is not widespread yet. Thus far, there is no experimental evidence that a fluorogenic substrate such as VRPR-AMC used in a metacaspase assay in *Arabidopsis *would be adapted for a proteolytic cell death in protozoa.

A protease able to bind the substrate FAM-VAD-FMK is present in *P. berghei *ookinetes (Figure 2D and 2E) but it should be noted that when assays were performed at 37°C (as per manufacturer's instructions) over 25% of these high-temperature-sensitive mosquito stages had disrupted membranes that were permeable to PI compared to approximately 12% if the assays were performed at 19°C. Incubation with the general caspase inhibitors Z-VAD-FMK and Boc-ASP-FMK and Z-DEVD-FMK, an inhibitor more specific to the caspase-3 subfamily, almost eliminated chromatin condensation in the parasite population. In contrast, incubation with Z-YVAD-CMK, a caspase-1 inhibitor, had no effect on chromatin condensation [17] nor did the cysteine protease clan CA inhibitors E64d or K11177, suggesting the parasite protease belongs to clan CD (Arambage and Hurd unpublished data). Further evidence that a cysteine protease is involved in ookinete cells death came from *in vivo *studies where feeding Z-VAD-FMK to mosquitoes during an infective blood meal significantly increased the intensity of infection [17]. However interpretation of *in vivo *experiments is complicated by the probability that inhibitors fed with the blood meal also inhibited the apoptosis occurring in midgut epithelial cells that have been traversed by ookinetes, with unknown effects on the induction of mosquito immune responses.

Two cysteine proteases have been annotated as metacaspase-like in the *P. falciparum *genome database. Incubation with Z-VAD-FMK resulted in a 75% decrease in the crisis forms of the parasite that has been proposed to be a form of apoptotic body [14]. Orthologues of PfMC1 were identified in several other species of *Plasmodium *and genetically modified *P. berghei *were produced in which the PbMC1 coding sequence had been removed (PbMC1-KO) [58]. Assays to compare markers for apoptosis in wild type and knock out parasites did not detect any significant differences in binding to the fluorochrome-labelled caspase inhibitor FAM-VAD-FMK. However, activation of caspase-like molecules in their wild type was extremely low (9.0% at 24 h) and they were unable to find any evidence of nuclear condensation or DNA fragmentation, and very few ookinetes were PS positive and PI negative. This is in marked contrast to the findings of Al-Olayan and colleagues [17,59] using the same *P. berghei *ANKA clone. Arambage and co-workers were, however, unable to detect any differences in the expression of apoptotic markers when this PbMCA1-KO was compared with the wild type, nor when comparisons between a PbMC2-KO and the wild type were made (Hurd, unpublished). Thus there is no sound evidence that the malaria metacaspase is associated with cell death, although there is some support for the view that cysteine proteases are related to the expression of other apoptotic markers.

A six-fold increase in the catalytic activity of a caspase-3-like protein was detected in bilirubin-treated erythrocyte stages of *P. falciparum *using the substrate DEVD-pNA and this activity was inhibited by Ac-DEVD-CHO, a potent inhibitor of caspase-3 [38]. The authors also detected a significant up-regulation of the expression of a putative apoptosis-like gene, *PfARP*, upon bilirubin treatment and both caspase-3-like activity and *PfARP *expression were prevented by antioxidants and ROS scavengers. The use of substrates and inhibitors to demonstrate a functional role for caspase-like molecules in malaria cell death and their use to pull out and identify these molecules warrants more study.

### Changes in the nucleus

Several changes to the nucleus have been described during the effector phase of apoptosis in metazoan cells. Together with DNA degradation, chromatin condensation and changes in nuclear morphology are often considered the best indicators of an apoptotic process. Those changes can be detected easily in higher eukaryotes by fluorescence microscopy using any of the several dyes that stain DNA in the nucleus. However, the small size of most of the protozoan parasites, and consequently of their nuclei, is an important drawback that has strongly restricted the use of this technique among parasitologists. Despite this difficulty, electron microscopy has been successfully used by some researchers to describe nuclear condensation, as can be seen in one of the two *Leishmania *promastigotes shown in Figure 1G (parasite in the right side of the picture). In *Trypanosoma brucei*, Welburn et al. [4] showed migration of chromatin to the periphery of the nuclear membrane while Figarella et al. showed that chromatin became equally distributed after incubation with prostaglandins from the J series [26].

Nuclear chromatin condensation and fragmentation has been detected in *P. berghei *ookinetes, as determined by fluorescence and electron microscopy [17,29,59]. The presence of nitric oxide donors in the culture medium increased the proportion of parasites exhibiting this marker [36]. Nuclear condensation was also observed to occur in bilirubin-treated *P. falciparum *intra-erythrocyte stages [38].

Beside other morphological changes (see above), condensed chromatin, particularly beneath the nuclear envelope, and nuclear pyknosis was also detected in *T. gondii *tachyzoites treated with SNP [10]. Importantly, these changes were accompanied by a time- and dose-dependent increase of parasites with hypoploid nuclei as determined by flow cytometry [10]. The latter quantitative analyses revealed that considerable amounts of SNP (actual concentrations of nitrate and nitrite as stable end products of NO turnover have not been measured) were required to induce high levels of hypoploid parasites, therefore the physiological relevance of this finding for the infection *in vivo *remains unclear. It nevertheless indicates that in *Toxoplasma*, several characteristic features, including nuclear changes, indicative of an apoptotic phenotype can occur.

Poly (ADP-ribose) polymerase (PARP) degradation is another characteristic marker of apoptosis frequently used in metazoans. PARP is a family of abundant nuclear proteins some of which are involved in the DNA base excision repair system, where they are potently activated following DNA strand nicks and breaks. In metazoans, the specific cleavage of PARP-1 by caspase-3/7 within the nuclear localization signal (^211^DEVD↓G^215^) generates two fragments of 24 and 89 kDa and this phenomenon has been used extensively as a biochemical marker of apoptosis. Some groups have described the use of monoclonal antibodies against PARP to detect PARP cleavage associated to cell death in *Leishmania *[22,60]. Unfortunately, whether the antibodies are raised against the mammalian or the parasite protein is not described in their manuscripts. Furthermore, in the genome database, there is no clear evidence of presence of protozoan PARP-1 encoding gene. Therefore, cleavage of PARP-1 as a marker of cell death in protozoan parasites still needs further investigation before being accepted as a marker.

DNA degradation is probably the most frequent marker of apoptotic death used in metazoans. In protozoans such as *Leishmania *it has been reported to occur after serum deprivation [34], heat shock [12], treatment with nitric oxide [61] or hydrogen peroxide [62] and with different drugs including pentostam [31], antimonials [63], novobiocin [22], miltefosine [23], edelfosine [46] or respiratory chain inhibitors [32].

In the beginning, analysis of DNA degradation in agarose gels became the easiest way to approach this process of cell death. During apoptosis, activated nucleases migrate to the nucleus to degrade DNA preferentially in the most accessible sequences: those located in the linker region of the nucleosomes, which generates the typical apoptotic DNA ladder. Whereas this methodology is widely used in research groups analyzing apoptosis in metazoans, several difficulties occur when these protocols were transferred to protozoans. In fact, some groups with vast experience in analysing apoptosis in human cells have been unable to adapt their protocols to detect a DNA ladder in parasites such as *Leishmania *(Mollinedo F. personal communication). Classical protocols are based on a simple direct purification of DNA from the cells by a phenol/chloroform extraction followed by ethanol precipitation or by incubation of the lysates with high NaCl concentration to disrupt nucleosomes and remove histones from DNA. A detailed analysis of the manuscripts that show clear DNA ladders in Trypanosomatids reveals that none of these "classical" protocols is used. Instead, most of them use a different method that includes an incubation of the samples with proteinase K. Alternatively, other protocols based on one specific commercial kit incubate chromatin for one hour at room temperature (even though the protocol recommends only 10 minutes) [64]. It must be stressed that these protocols only render clear oligonucleosomal ladders in samples obtained from dying parasites and not from healthy controls, which effectively indicates that one or several nucleases become active during the cell death process. On the other hand, DNA purified from the parasites without extracellular chromatin incubation shows death-induced degradation but without a clear pattern of oligonucleosomal laddering, which could indicate that the ladders are generated during the incubation of the extracted chromatin and not inside the parasites. In fact, Sen and co-workers reported that *in vitro *incubation of isolated nuclei from untreated *Leishmania *parasites with Ca^2+ ^and Mg^2+ ^at 37°C caused oligonucleosomal DNA degradation, a process that they named autodigestion [65]. Similarly, Zangger and co-workers found that no fragmentation could be detected in nuclei extracted from stationary phase parasites without any incubation process. Fragmentation was, however, visible following 30 minutes incubation and no increase in degradation was observed upon addition of Ca^2+ ^or Mn^2+^, while Mg^2+ ^seemed to increase the effect slightly [34]. In this latter study, DNA laddering was observed after isolation of the nuclei using a cavitation chamber. This experimental approach was instrumental to obtain a clear laddering. In conclusion, the two independent results obtained by Sen et al. and by Zangger et al. [34,65] reinforce the notion that a short period of incubation of the chromatin may be needed to obtain a clear laddering, which seems to explain the different results obtained by different groups depending on the protocols used.

In the last few years, the use of flow cytometry to determine the DNA content of the cells and detect the fraction of the population with DNA content lower than that characteristic of G1 cell cycle phase has been widely extended. Very simple protocols based on a mild permeabilization of the cells with ethanol and a short incubation of the permeabilized cells with RNAse in PBS with PI can be used [30]. Alternatively, non-permeabilized living cells can be directly stained with Hoechst 33342 and analyzed in a flow cytometer with an UV laser beam [37]. Experts in flow cytometry recommend analysing the fluorescence derived from propidium intercalation in the DNA in a linear scale and not in a logarithmic one.

Currently, DNA fragmentation revealed by the presence of a multitude of DNA strand breaks is considered to be the gold standard for identification of apoptotic cells. Several variants of the methodology that is based on fluorochrome-labeling of 3'-OH termini of DNA strand breaks in situ with the use of exogenous terminal deoxynucleotidyl transferase (TdT), commonly defined as the TUNEL assay, have been developed. Labelled cells can be analyzed by fluorescence microscopy or by flow cytometry, which can render a clear quantification of the percentage of labelled cells and the intensities of the fluorescence. The TUNEL approach was efficient in determining the percentage of dead *Leishmania *parasites in culture upon heat shock treatment [12], in their host cell [34] and ultimately, could be used to estimate the role of dead parasites enhancing the virulence of an inoculum [21,66]. Fragmentation of DNA was also detected in African trypanosomes in response to reactive oxygen species [67], incubation with PGJ2 [26] or the lectin ConA [6].

Fragmentation of genomic DNA from *Toxoplasma *into oligomers of nucleosomes became very evident after treatment of extracellular tachyzoites with SNP [10]. As already mentioned for the induction of hypoploid parasites (see above), treatment with high amounts of SNP for extended periods (20 hours) was required to detect a significant level of DNA laddering. In their protocol, the authors employed proteinase K and RNase A treatments suggesting that also in *Toxoplasma *alternative methods than those described for mammalian cells might be necessary to unequivocally detect DNA fragmentation. In addition to gel electrophoresis of genomic DNA oligonucleosomes, the TUNEL assay was also successfully employed to detect DNA strand breaks in *Toxoplasma *(Lüder, unpublished). In combination with flow cytometry it can be easily used to quantify the occurrence of DNA strand breaks in relatively large number of samples.

Following chloroquine treatment, Picot and colleagues detected the formation of DNA ladders in a population of the drug sensitive *P. falciparum*, 3D7, but not in the chloroquine resistant *P. falciparum *Lili strain. Conventional detection methodology was unsuccessful due, as suggested, to low sensitivity with small amounts of DNA. Their approach instead was 3'-OH end-labelling of extracted DNA with labelled nucleotides. Electrophoresis of samples was followed by Southern blotting and autoradiography [13]. DNA ladder formation was not detected following exposure of *P. falciparum *F32 strain to antimalarial parasite drugs when electrophoresis of DNA was followed by visualisation using ethidium bromide staining [39] or in the CSC-1 strain when SYBR Green 1 dye was used, where preliminary detection of DNA ladders was attributed to the presence of apoptotic white blood cells in the parasite culture [15]. These observations suggest the strategy adopted by Picot and co-workers may be required to detect DNA ladders in dead cells from the inter-erythrocytic stages of *P. falciparum*. However, in both of those latter studies drug treatments did not result in visualisation of other typical markers of apoptosis; a phenomenon that may be a strain effect related to drug sensitivity. Nyakeriga et al. [39] also failed to detect degradation of DNA into the larger sized structures associated with chromosome buttoning as a prelude to chromatin condensation using field inversion electrophoresis.

TUNEL was used to detect the increase in ookinetes containing nuclei with DNA fragmentation over time in culture (Figure 2F), even without the addition of stressors to the medium [29]. Following chloroquine (CQ) treatment the TUNEL assay was also used to detect a much higher proportion of DNA breaks in the CQ-sensitive 3D7 clone of *P. falciparum *than in the CQ-resistant 7G8 clone (over 50% compared with under 10%) [14]. Heat shock treatment (41°C) also induced approximately 60% of *P. falciparum *3D7 strain to be TUNEL positive [68]. However, no TUNEL positive cells were observed in F32 strain of *P. falciparum *following exposure to chloroquine, atovaquone or etoposide [39] and only a few were seen in the chloroquine resistant PSS1 strain when exposed to chloroquine, staurosporine or the nitric oxide donor *S*-nitroso-*N*-penicillamide (SNAP), even though loss of ΔΨ_m _had occurred [39].

DNA degradation in mammals is carried out by two nucleases, CAD and EndoG; CAD being the most active while EndoG seems to be especially relevant in caspase-independent apoptosis. Recently, three different groups have characterized nucleases similar to mammalian EndoG in different *Leishmania *species and also in *T. brucei *[69-71]. Two of them have also been able to show migration of these *Leishmania *EndoGs from the mitochondrion to the nucleus during the cell death process, either by using specific antibodies or by fusion of the protein to the green fluorescent protein (GFP) [69,71]. These antibodies or the chimaeric fusions could also be used as apoptotic markers in *Leishmania*.

## Conclusions

Despite the initial controversy, nowadays it is widely assumed that protozoan parasites are able to display some of the most characteristic apoptotic markers during cell death. The physiological role of the expression of apoptotic phenotypes in unicellular parasites has been questioned for many years but recent results demonstrate that this process is relevant, at least in some situations, for efficient progression of the parasite populations. For example, *in vitro *and *in vivo *infections of *Leishmania *promastigotes depend on the presence of PS positive parasites in the inoculums [21,66]. Similarly, Figarella et al. proposed that the stumpy forms of *T. brucei *produce prostaglandin D2, which induces cell death primarily of stumpy form cells [41]. This controlled process of cell death allows an efficient regulation of the size of the *T. brucei *population. The relevance of controlling cell size population in malaria parasites is discussed in another paper within this thematic series [72].

Typical markers of mammalian apoptosis have been widely used to characterize cell death in protozoans and, as shown in this review, many of the conventional assays for metazoans can be directly adapted for this purpose. However, several concerns have been raised for some of the most relevant markers. Regarding PS exposure, reviewers should be strict about the need to use double PS/PI staining and, preferably, at several time points. Similarly, probes used to analyze mitochondrial transmembrane potential in protozoans should initially be validated by the use of mitochondrial uncouplers. When studying protease activity, results derived from the use of caspase fluorogenic substrates or inhibitors should be analyzed with caution until the caspase-like activities observed in protozoa have been molecularly characterized. Moreover, the fact that metacaspases have already been demonstrated to be implicated in cell death in some protozoans should encourage the development of metacaspases-specific substrates as putative markers of this process. Regarding the protocols for DNA laddering in trypanosomatids, it must be stressed that ladders are only clearly observed when the nuclei are incubated in specific conditions after their purification. Finally, it may be important to consider that several protocols seem to perform better in protozoans when done at lower temperatures than those recommended for metazoans by kit manufacturers.

## List Of Abbreviations

**ΔΨ_m_**: Mitochondrial trans-membrane potential; **NO**: Nitric oxide; **NCCD**: Nomenclature Committee on Cell Death; **FSC**: Forward scatter; **SSC**: Side scatter; **SNP**: Sodium nitroprusside; **PS**: Phosphatidylserine; **PI**: Propidium Iodide; **FITC**: Fluorescein isothiocyanate; **ER**: Endoplasmic reticulum; **TMRM**: Tetramethylrhodamine methyl ester; **CCCP**: Carbonyl cyanide m-chlorophenylhydrazone; **FCCP**: Carbonyl cyanide p-trifluoromethoxy-phenylhydrazone); **DEVD-FMK**: Asp(OMe)-Glu(OMe)-Val-Asp(OMe)-Fluoromethylketone; **PARP**: Poly (ADP-ribose) polymerase; **JC-1**: 5,5',6,6'-tetrachloro-1,1',3,3'-tetraethylbenzimidazolylcarbocyanine iodide; **TdT**: Terminal deoxynucleotidyl transferase; **CQ**: Chloroquine; **TUNEL**: Terminal Transferase dUTP Nick End Labeling; **SNAP**: *S*-nitroso-*N*-penicillamide; **GFP**: Green fluorescent protein; **DiOC_6_**: 3,3'-dihexyloxacarbocyanine iodide.

## Competing interests

The authors declare that they have no competing interests.

## Authors' contributions

AJ drafted and coordinated the contributions of the different authors to the manuscript and specifically contributed with the information related to the *Leishmania *parasite. JFA collected data, helped to draft the information related to *Leishmania *and critically reviewed the manuscript. EM collected data, helped to draft the information related to *Trypanosoma *and critically reviewed the manuscript. CL collected data, helped to draft the information related to *Toxoplasma *and critically reviewed the manuscript. NF collected data, helped to draft the information related to *Leishmania *and critically reviewed the manuscript. HH collected data, helped to draft the information related to *Plasmodium *and critically reviewed the manuscript.
